# 6*-*Bromohypaphorine from Marine Nudibranch Mollusk *Hermissenda crassicornis* is an Agonist of Human α7 Nicotinic Acetylcholine Receptor

**DOI:** 10.3390/md13031255

**Published:** 2015-03-12

**Authors:** Igor E. Kasheverov, Irina V. Shelukhina, Denis S. Kudryavtsev, Tatyana N. Makarieva, Ekaterina N. Spirova, Alla G. Guzii, Valentin A. Stonik, Victor I. Tsetlin

**Affiliations:** 1 Shemyakin-Ovchinnikov Institute of Bioorganic Chemistry, Russian Academy of Sciences, Miklukho-Maklaya Street, 16/10, Moscow 117997, Russia; E-Mails: iekash@mx.ibch.ru (I.E.K.); ner-neri@mail.ru (I.V.S.); kudryavtsev@ibch.ru (D.S.K.); katya_spirova@mail.ru (E.N.S.); 2 G.B. Elyakov Pacific Institute of Bioorganic Chemistry (PIBOC), Russian Academy of Sciences, Prospect 100 let Vladivostoku, 159, Vladivostok 690022, Russia; E-Mails: makarieva@piboc.dvo.ru (T.N.M.); gagry@rambler.ru (A.G.G.); stonik@piboc.dvo.ru (V.A.S.)

**Keywords:** 6*-*bromohypaphorine, nicotinic acetylcholine receptors, agonist

## Abstract

6*-*Bromohypaphorine (6-BHP) has been isolated from the marine sponges *Pachymatisma johnstoni*, *Aplysina* sp., and the tunicate *Aplidium conicum*, but data on its biological activity were not available. For the nudibranch mollusk *Hermissenda crassicornis* no endogenous compounds were known, and here we describe the isolation of 6-BHP from this mollusk and its effects on different nicotinic acetylcholine receptors (nAChR). Two-electrode voltage-clamp experiments on the chimeric α7 nAChR (built of chicken α7 ligand-binding and glycine receptor transmembrane domains) or on rat α4β2 nAChR expressed in *Xenopus* oocytes revealed no action of 6-BHP. However, in radioligand analysis, 6-BHP competed with radioiodinated α-bungarotoxin for binding to human α7 nAChR expressed in GH_4_C_1_ cells (IC_50_ 23 ± 1 μM), but showed no competition on muscle-type nAChR from *Torpedo californica*. In Ca^2+^-imaging experiments on the human α7 nAChR expressed in the Neuro2a cells, 6-BHP in the presence of PNU120596 behaved as an agonist (EC_50_ ~80 μM). To the best of our knowledge, 6-BHP is the first low-molecular weight compound from marine source which is an agonist of the nAChR subtype. This may have physiological importance because *H. crassicornis*, with its simple and tractable nervous system, is a convenient model system for studying the learning and memory processes.

## 1. Introduction

Marine invertebrates are a rich source of bioactive secondary metabolites with fascinating chemical structures and biological activities [1]. In many cases such metabolites play a defensive role for those organisms and may be considered as potential drug leads for anticancer agents and also for other medical applications [2]. For some of these metabolites, new biological targets were identified, opening new ways to decipher the mechanisms of action. In particular, several low-molecular weight compounds from marine invertebrates have recently been shown to bind and inhibit the nicotinic acetylcholine receptors (nAChRs) [3].

In the present work, our attention was focused on the marine nudibranch mollusk *Hermissenda crassicornis* from Troitsa Bay in the Sea of Japan, for the following reasons. First of all, this mollusk is used as a convenient model system for studying the cellular and molecular processes that underlie learning and memory capacities [4]. Although there is no information about biologically active compounds present in this organism, it might contain some substances playing a role in endogenous modulation of its nervous system, or being involved in defensive mechanisms. Thus, we wanted to isolate those putative compounds and to check if they can interact with different types of nAChRs. In a first attempt we managed to isolate only one compound for which we have determined the chemical structure. It happened to be 6*-*bromohypaphorine (6-BHP), which was earlier isolated from the sponge *Pachymatisma johnstoni* [5]. However, in spite of various activities described for its close analog 5,6-dibromohypaphorine [6] or for other brominated indole derivatives [2], no tests of activity have been reported for 6*-*BHP. Here, we demonstrate that 6*-*BHP can distinguish several subtypes of nAChRs, namely by showing no binding to the muscle-type nAChR from the electric organ of the *Torpedo californica* ray and not affecting the acetylcholine-induced currents in the *Xenopus* oocytes expressed heteromeric rat α4β2 nAChR or chimeric chicken α7 nAChR, but binding with a micromolar affinity to human α7 nAChR and playing that role as an agonist.

## 2. Results and Discussion

### 2.1. Isolation and Structure Determination for 6-Bromohypaphorine from Hermissenda crassicornis

**Figure 1:**
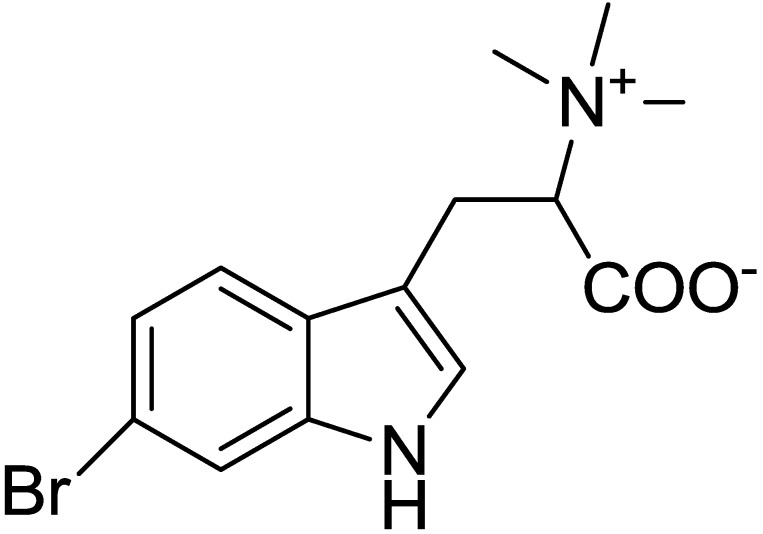
Chemical structure of l-6-Bromohypaphorine (6-BHP).

l-6-Bromohypaphorine (6-BHP) was isolated from the fresh nudibranches by extraction with EtOH, partition between H_2_O and n-BuOH, then BuOH-soluble materials were partitioned between aqueous EtOH and hexane, and column chromatography of the ethanol soluble materials was performed on a reversed phase YMC*Gel ODS-A column (YMC Co., Ltd., Kyoto, Japan) using a gradient EtOH/H_2_O. The structure of the compound (Figure 1) was established using the detailed analysis of 1D and 2D NMR, mass spectra, optical rotation data, and by comparison with literature data.

### 2.2. Two-Electrode Voltage-Clamp

The experiments were performed on heterologously expressed individual subtypes of nAChRs: Chimeric chicken α7 nAChR/GlyR (built of chicken α7 ligand-binding domain and transmembrane portion of glycine receptor) or on rat α4β2 nAChR. The plasmids with respective cDNAs were introduced into *Xenopus laevis* oocytes and the ion currents were measured after two to three days.

A typical experiment consisted of consecutive application, to oocyte, of acetylcholine or epibatidine solution, 6-BHP solution in pure buffer, 6-BHP in buffer solution with addition of acetylcholine or epibatidine. Specific antagonists, α-cobratoxin and dihydro-β-erythroidine (DHβE) (in case of α7/GlyR and α4β2 nAChR, respectively), were applied after all recordings to each oocyte to validate target receptor expression.

**Figure 2:**
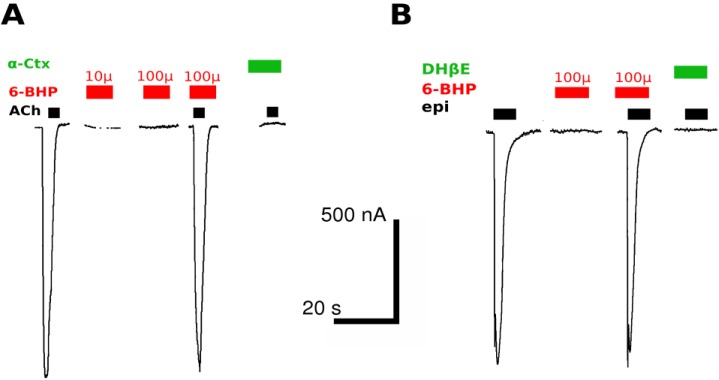

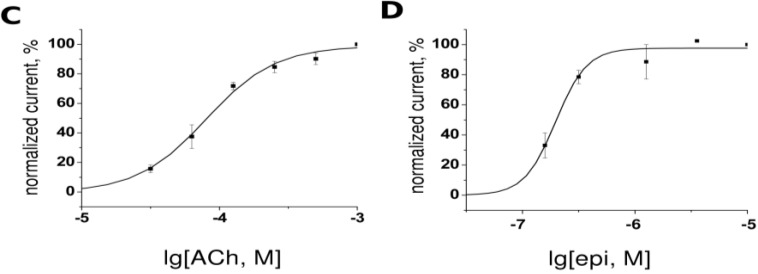
(**A**) Oocytes expressing chicken α7/GlyR chimera respond to application of 100 μM acetylcholine (ACh, black bars) but not to 10 or 100 μM 6-BHP (red bars). 6-BHP at 100 μM also did not inhibit acetylcholine-evoked current, while α-cobratoxin at 1 μM completely abolished it (α-CTX, green bar); (**B**) Oocytes expressing rat α4β2 nAChR respond to application of 10 μM epibatidine (epi, black bars) but not 100 μM 6-BHP (red bars), which at concentrations up to 100 μM did not inhibit epibatidine-evoked current, while DHβE at 1 μM completely abolished it (green bar). Bars show scale for time and current; (**C**) Dose-response curve for acetylcholine at chimeric α7/GlyR (EC_50_ = 80 ± 5 μM, *n* = 3–4); (**D**) Dose-response curve for epibatidine at α4β2 nAChR (EC_50_ = 190 ± 10 nM, *n* = 3–4).

6-BHP failed to evoke a current through chicken α7 nAChR/GlyR chimera or rat α4β2 nAChR (Figure 2). Additionally, no inhibition of the agonist-evoked current was detected with 6-BHP at concentrations of up to 100 μM.

### 2.3. Radioligand Analysis

**Figure 3:**
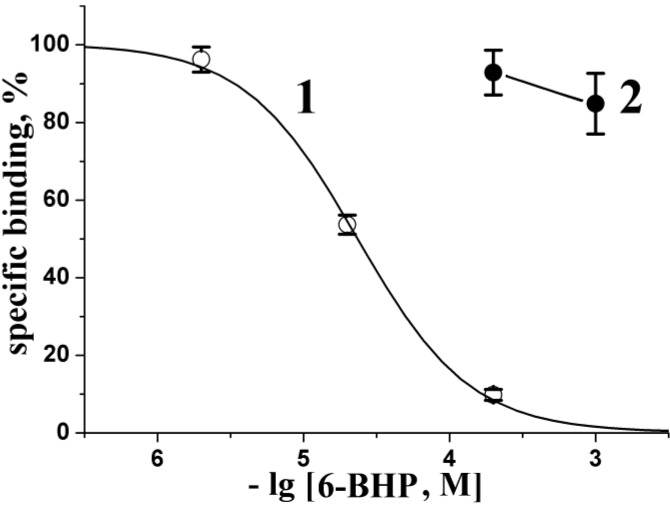
Inhibition of initial rate for radioiodinated α-bungarotoxin ([^125^I]-αBgt) binding to human α7 nAChR (1, open circles) or muscle-type nAChR from *T. californica* (2, filled circles) with 6-BHP. Each point is a mean ± s.e.m. value of three measurements for each concentration in two independent experiments. The curve (1) for α7 nAChR expressed in the GH_4_C_1_ cells was calculated from the means ± s.e.m. using the ORIGIN 7.5 program (see Experimental Section). The respective IC_50_ = 23 ± 1 μM. Virtually no evident inhibition of [^125^I]-αBgt binding to *T. californica* nAChR was detected with 6-BHP at concentrations up to 1000 μM.

The affinity of 6-BHP for agonist/competitive antagonist binding sites of neuronal and muscle-type nAChRs was evaluated in competition with [^125^I] iodinated α-bungarotoxin for binding to human α7 nAChR transfected in GH_4_C_1_ cells and to nAChR from the *T. californica* ray electric organ, respectively. 6-BHP did not show any appreciable inhibitory activity on muscle-type receptor even at a concentration of 1000 μM (Figure 3). On the other hand, it completely blocked, with IC_50_ 23 ± 1 μM (Figure 3), the binding of radioligand to human α7 nAChR in GH_4_C_1_ cells, exhibiting distinct selectivity for this receptor subtype.

### 2.4. Calcium Imaging

**Figure 4:**
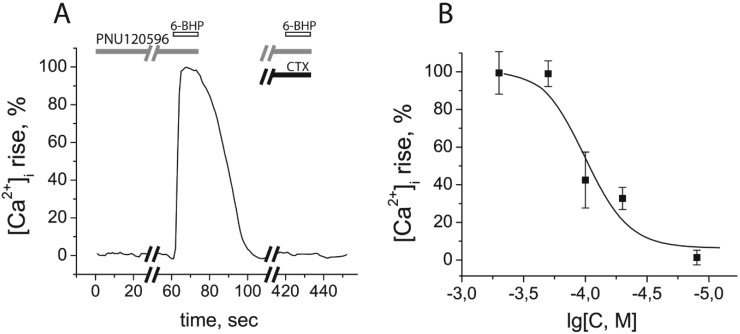
6-Bromohypaphorine (6-BHP) from *Hermissenda crassicornis* provoked an intracellular calcium concentration rise ([Ca^2+^]_i_ rise) in neuroblastoma Neuro2a cells expressing human α7 nAChR. (**A**) Normalized [Ca^2+^]_i_ rise after application of 200 μM 6-BHP and its inhibition by 2-min preincubation with 2 μM α-cobratoxin (CTX), a specific antagonist of α7 nAChR; (**B**) A curve of [Ca^2+^]_i_ rise in response to different concentrations of 6-BHP. All ligand solutions contained 10 μM PNU120596, a positive allosteric modulator of α7 nAChR. The calculated EC_50_ value was 82.7 ± 20.1 μM (mean ± s.e.m., *n* = 5).

Radioligand analysis showed that 6-BHP competitively binds to human α7 nAChR in the micromolar range. To find out if 6-BHP is an agonist or antagonist of α7 nAChR, calcium-imaging experiments were performed. Detection of small and fast-decaying calcium responses in α7 nAChR is difficult and, to solve this problem, we amplified those responses by adding PNU120596 (at 10 μM), a selective positive allosteric modulator of α7 nicotinic receptor, to all ligand solutions. Binding to an allosteric intra-subunit trans-membrane site, this compound increases both the magnitude and duration of agonist-evoked ion flux [7,8,9,10]. Under these conditions, 6-BHP initiated an intracellular calcium concentration rise in the neuroblastoma Neuro2a cells, transiently expressing human α7 nAChR (Figure 4, EC_50_ = 82.7 ± 20.1 μM (mean ± s.e.m.)). This calcium response was completely inhibited by a selective α7 nAChR antagonist α-cobratoxin (CTX, Figure 4A), confirming the specificity of 6-BHP agonistic action on α7 nAChR subtype.

### 2.5. Discussion

It is interesting to compare our results for 6-BHP with the literature data on the activity of its closest homolog, namely 5,6-dibromohypaphorine, as well as with some other compounds containing brominated indole rings. As mentioned in the review [2], diverse compounds isolated from marine sources, namely 5,6-dibromotryptamine, 5,6-dibromo-*N*-methyltryptamine, 5,6-dibromo-*N*-methyltryptophan (dibromoabrine), 5,6-dibromo-*N*,*N*-dimethyltryptamine, and 5,6-dibromohypaphorine have shown anti-cancer and anti-inflammatory properties. For us, most interesting are the activities implying, if not the interactions with the nAChRs (we could not find such data), at least with some receptors or ion channels. In fact, indirect data indicate that such interactions are possible. Hypaphorine, an indole alkaloid secreted by the fungus *Pisolithus microcarpus*, was shown to increase cytosolic calcium concentration and to inhibit root hair tip growth due to a transient depolarization of the plasma membrane and reorganization of the actin and microtubule cytoskeletons [11]. The interaction of hypaphorine with a putative receptor or ion channel may be anticipated because it was shown to induce sleep in mice [12]. In this respect, another compound, inducing a sleep activity and containing a brominated amino acid (in fact, several brominated tryptophan residues), should be mentioned, namely a 33-residue peptide from the poisonous fish-hunting marine snail *Conus radiatus* [13]. It is noteworthy that, in addition to bromination, other kinds of post-translational modifications (phosphorylation, glycosylation and some other, see reviews [14,15]) were detected in various peptides from *Conus* venoms.

One more effect described for hypaphorine is its inhibition of indole-3-acetic acid (IAA) activity in seedling roots, which might result from the competition for the interaction with auxin-binding proteins [16]. A possibility of competition with IAA for binding to horseradish peroxidase was also discussed [17].

However, for hypaphorine, we could not find direct evidence for interaction, at the molecular level, with another target. Conicamin, which resembles hypahorine, but contains neither a bromine atom in the indole ring nor a carboxylic group and incorporates double bond in the chain attaching the tertiary amine, was shown to be a histamine antagonist [18]. An appropriate example of an interaction, characterized at the molecular level, is binding at low micromolar concentration of a cyclic dipeptide containing 6-bromotryptophan residue to the two subtypes of metabotropic serotonin receptors [19]. Thus, the 6-BHP interaction with the identified target, namely with the human α7 nAChR, is the first described for hypaphorine and its brominated analogs. It is not yet clear whether this sort of interaction might be important for the activity and/or protection of *Hermissenda crassicornis* or for those organisms where 6-BHP was found earlier (*Pachymatisma johnstoni* [5], *Aplidium conicum* [18], and *Aplysina* sp. [20]). However, in view of the human α7 nAChR implication in diverse neurodegenerative diseases, the discovered agonistic activity of 6-BHP can be a hint for the development of new drugs.

The described interaction of 6-BHP, a compound containing the indole ring, with the α7 nAChR can be compared with the interactions of other indole-comprising compounds, such as serotonin and its analogs, with distinct nAChR subtypes. Serotonin (5-hydroxytryptamine) is a neurotransmitter acting, not only through several subtypes of metabotropic 5HT receptors, but also through the 5HT-3 receptor, an ion channel belonging to the same family of Cys-loop receptors as nAChRs (see review [21]). Interestingly, at millimolar concentrations, serotonin was shown to compete with α-bungarotoxin for binding to *Torpedo* nAChR [22] and to decrease the current amplitudes in the mouse muscle nAChR [23]. With much higher affinity, at micromolar concentrations, serotonin inhibits neuronal α9α10 nAChRs [24]. Another facet of this receptor-ligand cross-talk is the inhibition of the 5HT-3 receptor by d-tubocurarine and its analogs [25], well-known antagonists of various subtypes of nAChRs. In all these cases the inhibitory effect was registered, but a recent publication demonstrated that vareniclin, a partial agonist of α4β2 nAChRs, is also agonist of the human 5HT-3 receptor [26]. In view of all these examples we plan a more detailed analysis of 6-BHP interactions with different nAChR subtypes and other probable receptor targets. Of particular interest would be elucidation of why 6-BHP is an agonist of human α7 nAChRs but does not recognize the chimeric receptor comprising the ligand-binding domain of the chicken α7 subunit. The answer may be found after a detailed comparison of the human/chicken α7 sequences and a subsequent analysis of interactions with the chosen mutants. It is appropriate to mention here that α-conotoxin RgIA acts with a very large difference in affinity (over two orders of magnitude) on rat and human α9α10 nAChRs [27], thus that against the human receptor it can be conventionally considered as “inactive”. Surprisingly, a single mutation Thr56Ile is the reason for this difference [27].

## 3. Experimental Section

### 3.1. Isolation and Structure Determination of l-6-Bromohypaphorine

#### 3.1.1. Animal Material and Isolation of 6-BHP

About 200 specimens of *Hermissenda crassicornis* (average size 10 mm) were collected in August 2014 from Troitsa Bay, in the Sea of Japan (42°37′333ʺ N; 131°07′50ʺ E, depth 10 m). Animal material was immediately extracted by EtOH. 6-BHP (2.5 mg) was isolated from *H. crassicornis* after extraction with EtOH, evaporation, partition between H_2_O and n-BuOH, partition BuOH-soluble materials between aqueous EtOH and hexane, and column chromatography of the ethanol soluble materials on a reversed phase YMC*Gel ODS-A column (YMC Co., Ltd., Kyoto, Japan) using a gradient EtOH/H_2_O.

#### 3.1.2. Physical Characteristics and Spectral Data fo 6-BHP

l-6-Bromohypaphorine: Amorpous solid; [α]_D_ = +50 (c 0.25, EtOH), lit. data: [α]_D_ = +58 (MeOH/TFA, 8:1) [5]; ^1^H NMR (CD_3_OD, 700 MHz) δ 7.54 (1H, d, *J* = 8.6, H-4), 7.49 (1H, d, *J* = 1.5, H-7), 7.19 (1H, s, H-2), 7.14 (1H, dd, *J* = 1.5, 8.6, H-5), 3.85 (1H, t, *J* = 7.4, H-9), 3.40 (2H, d, *J* = 7.4, H_2_-8), 3.29 (9H, s, N(CH_3_)_3_); ^13^C CMR (CD_3_OD, 175 MHz) δ 172.1 (C-10), 139.5 (C-7a), 127.9 (C-3a), 126.7 (C-2), 123.8 (C-5), 121.3 (C-4), 116.6 (C-6), 115.9 (C-7), 110.1 (C-3), 81.1 (C-9), 53.3 (N(CH_3_)_3_), 25.0 (C-8); HRESIMS *m*/*z* 649/651/653, *m*/*z* 649.1015 [2M + H]^+^ (calculated for C_28_H_35_O_4_N_4_^79^Br_2_, 649.1020), *m*/*z* 347/348/349, *m*/*z* 347.0368 [M + Na]^+^ (calculated for C_14_H_17_O_2_N_2_^79^BrNa, 347.0366). Optical rotations were measured using a Perkin-Elmer 343 polarimeter. The ^1^H and ^13^C NMR spectra were recorded on Avance III-700 spectrometers at 700 and 175 MHz, respectively, and chemical shifts were referenced to the corresponding residual solvent signal (δH 3.30/δC 49.60 for CD3OD). ESI mass spectra (including HR ESI-MS) were obtained on an Agilent 6510 Q-TOF LC-MS spectrometer by direct injection in MeOH.

### 3.2. Heterologous Expression of the nAChR Subtypes in Xenopus Oocytes and Electrophysiology Measurements

Recordings were performed using turbo TEC-03X amplifier (npi electronic, Tamm, Germany) and WinWCP recording software (University of Strathclyde, Glasgow, UK) on oocytes removed from mature Xenopus frogs. Two to three days before recordings, oocytes were injected with plasmid DNA, containing α4, β2 nAChR (*Rattus* sp.) or chicken α7 nAChR/GlyR chimera and kept at 18 °C in ND96 electrophysiology buffer solution (5 mM HEPES/NaOH at pH 7.6 and 18 °C, 96 mM NaCl, 2 mM KCl, 1.8 mM CaCl_2_, 2 mM MgCl_2_). For details on α7 nAChR/GlyR chimera, see [28].

### 3.3. Heterlogous Expression of the α7 nAChR in Neuroblastoma Neuro2a Cell Line and Ca^2+^ Measurements in Response to Agonists and Antagonists

Mouse neuroblastoma Neuro2a cells were transiently transfected with plasmids α7 nAChR-pCEP4, Ric3-pCMV6-XL5 (OriGene, Rockville, MD, USA) and pCase12-cyto vector (Evrogen, Moscow, Russia) following lipofectamine transfection protocol (Invitrogen, Waltham, MA, USA). Shaperone Ric3 significantly increased human α7 nAChR expression level in Neuro2a cells. Transfected cells were grown in DMEM (Paneco, Moscow, Russia) supplemented with 10% fetal bovine serum (PAA Laboratories, Austria) at 37 °C in CO_2_-incubator for 72 h.

The intracellular calcium concentration [Ca^2+^]_i_ measurements were performed in external buffer containing 140 mM NaCl, 2 mM CaCl_2_, 2.8 mM KCl, 4 mM MgCl_2_, 20 mM HEPES, 10 mM glucose, pH 7.4 at room temperature. Expression of Case12, a fluorescent genetically encoded sensor of calcium ions (ex/em = 491/516 nm), allowed a direct monitoring of changes in [Ca^2+^]_i_ using an epifluorescent microscope (Olympus, Japan) with an appropriate filter combination. The cells were exposed to 40 μM acetylcholine, 12.5–500 μM 6-BHP, and 2 μM α-cobratoxin solutions, and changes in Case12 fluorescence were recorded from each cell independently. To increase the registered changes, all ligand solutions contained α7 nAChR positive allosteric modulator PNU120596 (10 μM, Tocris, Bristol, UK).

### 3.4. Radioligand Assay

In competition experiments with [^125^I]-αBgt, 6-BHP (in concentration range of 2–1000 μM) was pre-incubated 2 h at room temperature with the GH_4_C_1_ cells (6.5 μg of total protein with final concentration of 0.4 nM of toxin-binding sites) or *Torpedo californica* electric organ membranes (final concentration 1.25 nM of toxin-binding sites) in 50 μL of binding buffer (20 mM Tris-HCl buffer, 1 mg/mL of bovine serum albumin, pH 7.8). After that [^125^I]-αBgt was added to GH_4_C_1_ cells or membranes to final concentration 0.2 nM and the mixtures were additionally incubated for 5 min. Binding was stopped by rapid filtration on GF/C filters (Whatman, Little Chalfont, UK) pre-soaked in 0.25% polyethylenimine, unbound radioactivity being removed from the filters by washout (3 × 3 mL) with the binding buffer. Non-specific binding was determined in all cases using 2 h pre-incubation with 20 μM α-cobratoxin.

The binding results were analyzed using ORIGIN 7.5 (OriginLab Corporation, Northampton, MA, USA) fitting to a one-site dose-response curve by the following equation:

% response = 100/{1 + ([toxin]/IC_50_)*^n^*}

where IC_50_ is the concentration at which 50% of the binding sites are inhibited and *n* is the Hill coefficient.

## 4. Conclusions

In summary, we present here the activity of the first compound isolated from the marine nudibranch mollusk *Hermissenda crassicornis*. Its structure is identical to that of l-6-bromohypophorine (6-BHP) for the first time isolated over three decades ago from the marine sponges *Pachymatisma johnstoni* [5], later from *Aplysina* sp. [20] and the tunicate *Aplidium conicum* [18]. However, since that time there were no data about the activity of 6-BHP. We found that it interacts quite efficiently with human α7 nAChR, being inactive toward either another subtype of neuronal nAChR, namely α4β2 nAChR, or against muscle-type nAChR from *Torpedo californica* ray. No inhibition of ion currents was observed with 6-BHP applied to the chimeric receptor comprising the ligand-binding domain of chicken α7 nAChR. These first results on the specificity of 6-BHP can be compared with the properties of snake venom neurotoxins and α-conotoxins from *Conus* marine snails. In the neurotoxin family, short α-neurotoxins block only muscle-type nAChRs, long α-neurotoxins inhibit both muscle-type and homooligomeric neuronal nAChRs (like the α7 and α9 ones), while κ-bungarotoxin is effective against heteromeric α3β2 nAChRs (see review [14]). Species specificity is manifested most clearly with denmotoxin from the colubrid snake venom, which blocks, quite potently, chicken muscle nAChR and only weakly that of mouse [29]. In terms of nAChR subtype specificity, the leaders are α-conotoxins, which not only discriminate muscle-type from neuronal nAChRs, but with the aid of naturally-occurring α-conotoxins and their synthetic analogs, virtually all subtypes of neuronal nAChRs can be identified and selectively blocked (see reviews [14,15]). Most interestingly, it seems that 6-BHP not only recognizes the human α7 nAChR, but it acts as an agonist. In this paper we also presented a brief review of the activities of different bromo-containing compounds of marine origin and demonstrated that, for such a family, the 6-BHP interaction with the human α7 nAChR is among those rare cases when this activity is characterized at the molecular level.
